# Serial dual-wavelength illumination for retinal vessel oximetry using a conventional fundus camera: a proof-of-concept study

**DOI:** 10.1186/s12886-026-05216-7

**Published:** 2026-08-08

**Authors:** Melih Tarhan, Daniel Meller, Martin Hammer

**Affiliations:** 1 Department of Ophthalmology, University Hospital Jena, Am Klinikum 1, 07747 Jena, Germany; 2 Center of Medical Optics and Photonics, Friedrich Schiller University Jena, Jena, Germany

**Keywords:** Retinal vessel oximetry, Fundus camera, Dual-wavelength imaging, Serial illumination, Oxygen saturation, Retinal imaging, Optical density ratio, Spectrophotometry

## Abstract

**Purpose:**

Retinal vessel oximetry requires fundus images at two wavelengths with different hemoglobin absorption characteristics. Simultaneous acquisition using one-chip color cameras may be affected by incomplete spectral separation of detector channels, whereas multi-chip systems such as 3-chip CCD cameras can achieve improved spectral separation. We present a serial dual-wavelength illumination approach integrated into a conventional fundus camera and compare oxygen saturation measurements with those obtained from a commercial retinal oximeter.

**Methods:**

A custom LED illumination unit was fiber-coupled into a fundus camera to provide serial illumination at 548 nm (isosbestic hemoglobin wavelength) and 605 nm (oxygen-sensitive wavelength with differential absorption between oxy- and deoxyhemoglobin), each for 250 ms. Corneal irradiance was 33 mW/cm² at 548 nm and 7 mW/cm² at 605 nm. Sequentially acquired images were registered and analyzed using established retinal oximetry algorithms. Mean arterial and venous oxygen saturation values were compared with measurements from a commercial retinal oximeter in nine participants undergoing routine ophthalmic examination. Linear regression analysis was performed to assess the association between methods, and agreement was additionally evaluated using Bland–Altman analysis.

**Results:**

Mean arterial oxygen saturation was 99.4 ± 5.7% with serial illumination and 100.4 ± 3.0% with standard oximetry. Mean venous oxygen saturation was 71.5 ± 7.0% and 69.5 ± 11.6%, respectively. The mean absolute differences between both techniques were 3.6 ± 2.7% for arteries and 8.4 ± 9.3% for veins. Bland–Altman analysis showed a small arterial bias of 0.94% with limits of agreement from − 7.9% to + 9.8%. For venous measurements, bias was − 1.98% with wider limits of agreement (− 26.9% to + 22.9%). Arterial measurements showed borderline non-significant correlation (*R* = 0.606, *p* = 0.084). Venous measurements showed no correlation.

**Conclusions:**

Serial illumination is feasible for retinal vessel oximetry and mitigates detector-level spectral crosstalk associated with simultaneous dual-wavelength imaging using one-chip color cameras. Arterial measurements showed only a small systematic bias compared with the commercial retinal oximeter, whereas venous measurements demonstrated greater variability, indicating the need for further optimization and validation.

## Introduction

The retina is among the most metabolically active tissues, and retinal oxygen delivery and extraction are tightly regulated by autoregulatory vascular mechanisms [1–3]. Disturbances of retinal oxygenation are implicated in major ocular diseases such as diabetic retinopathy, retinal vascular occlusions, glaucoma, and age-related macular degeneration [4–6]. Retinal vessel oximetry offers a non-invasive optical approach to estimate hemoglobin oxygen saturation in retinal arteries and veins based on wavelength-dependent absorption differences of oxyhemoglobin and deoxyhemoglobin [7, 8].

Fundus-camera-based retinal oximetry commonly uses dual-wavelength reflectance imaging and derives saturation values from optical density ratios, typically including compensation for vessel diameter and fundus pigmentation to reduce bias and variability [9–11]. Historically, improved spectral separation in retinal oximetry has been achieved using multi-chip camera systems, such as 3-chip CCD cameras, in which the red, green, and blue signals are detected by separate sensors. In contrast, most conventional fundus cameras employ one-chip color sensors with Bayer filter arrays, whose detector channels exhibit partially overlapping spectral sensitivities. Consequently, simultaneous dual-wavelength acquisition with such cameras can result in imperfect wavelength separation (crosstalk) and thereby distort optical density estimates [8, 10]. Although algorithmic compensation may mitigate some confounding factors, it cannot fully eliminate detector-intrinsic spectral overlap [8, 10]. Dedicated retinal imaging systems employing monochrome detectors together with wavelength-selective optical filtering or hyperspectral imaging approaches can largely avoid detector-level spectral crosstalk by providing high spectral selectivity [12, 13]. However, such systems require dedicated optical hardware and are not directly compatible with conventional color fundus cameras. Commercial retinal oximetry systems may employ narrow dual-bandpass filters. When such filters are combined with conventional one-chip Bayer color cameras, however, the partially overlapping spectral sensitivities of the detector channels may still contribute to spectral crosstalk. Serial dual-wavelength illumination addresses this limitation by shifting wavelength separation from the detection to the illumination stage.

Serial illumination therefore provides an alternative approach for retinal oximetry implemented on conventional color fundus cameras. This approach avoids detector-level crosstalk while using conventional color-camera hardware and without requiring monochrome detectors or external filter assemblies. The concept is consistent with the spectrophotometric principles of retinal oximetry [7, 8] and resembles sequential approaches used in other multispectral retinal imaging contexts [12, 14]. Similar nonsimultaneous dual-wavelength acquisition approaches for retinal oximetry using conventional fundus cameras have previously been described by Kim et al. [15] and Singh et al. [16].

Building on these approaches, the present work evaluates serial dual-wavelength illumination as a strategy to mitigate detector-level spectral crosstalk by shifting wavelength separation from the detection to the illumination stage. This is implemented using a fiber-coupled LED illumination system integrated into a clinical fundus camera platform, enabling flexible wavelength control without mechanical filter exchange. Inter-method agreement with a commercially available retinal oximeter is quantitatively assessed using Bland–Altman analysis, complemented by linear regression analysis to evaluate the association between methods. The key trade-off of serial acquisition is increased sensitivity to inter-frame eye motion, requiring image registration and careful timing [14].

Here we implemented serial dual-wavelength LED illumination in a conventional fundus camera and compared oxygen saturation measurements with a commercial retinal oximeter. Because arterial and venous measurements differ in robustness and variability in retinal oximetry research, we present and discuss them separately.

## Methods

### Study population and ethics

Nine participants undergoing routine ophthalmic examination were enrolled in this exploratory study. The study cohort comprised nine participants (5 male, 4 female) with a mean age of 64.7 ± 15.9 years (range: 41–89 years). All participants were of European descent. Inclusion criteria comprised the ability to undergo standard fundus photography and the absence of media opacities severely affecting image quality. Exclusion criteria included known retinal diseases, glaucoma, diabetes mellitus, and diseases of the cardiovascular system, as these conditions may influence retinal oxygenation and vascular physiology. The study adhered to the Declaration of Helsinki and was approved by the local institutional ethics committee. Written informed consent was obtained from all participants prior to examination.

### Serial illumination setup

A custom LED illumination unit was fiber-coupled into a fundus camera (Visucam 500, Carl Zeiss Meditec AG, Jena, Germany). Sequential illumination was performed at 548 nm (isosbestic hemoglobin wavelength) and 605 nm (oxygen-sensitive wavelength with differential absorption between oxy- and deoxyhemoglobin) with a bandwidth of 2.5 nm, each for 250 ms, resulting in 500 ms total exposure time per dual-wavelength pair. Corneal irradiance was 33 mW/cm² at 548 nm and 7 mW/cm² at 605 nm. Based on corneal irradiance, ocular transmission characteristics, and retinal image geometry, retinal exposure was estimated to remain below the maximum permissible exposure (MPE) limits for both photochemical and thermal retinal hazards as defined in ISO 15004-2 for ophthalmic instruments.

### Image acquisition and processing

Fundus images were acquired sequentially at both wavelengths. Subjects were not dark-adapted but were examined under ambient room illumination. Pupils were dilated using topical tropicamide (Mydriaticum Stulln^®^ UD, Pharma Stulln GmbH, Stulln, Germany). One image was acquired at each wavelength. A 30° field centered on the optic disc was imaged. Image registration was performed using customized software based on affine transformations. Registration quality was assessed by visual inspection of vessel alignment and vessel-edge correspondence between the registered image pairs. No image repetition was required because all acquired image pairs were of sufficient quality for analysis. Registered image pairs were subsequently analyzed for oxygen saturation.

### Oxygen saturation calculation

Oxygen saturation was derived from optical density ratios obtained at 548 nm and 605 nm using an established dual-wavelength retinal oximetry algorithm [11]. The calculation incorporated compensation of the oxygen-saturation estimate for vessel diameter and fundus pigmentation [10]. Fundus pigmentation correction was performed according to the previously published calibration procedure [10]. Vessel diameter was determined from the intensity profile perpendicular to the vessel axis using the previously described retinal vessel profile analysis method [17]. Oxygen saturation derived from the optical density ratio was subsequently corrected for vessel diameter according to the previously published calibration and compensation procedure [10]. Calibration of the serial illumination system was performed independently of the commercial retinal oximeter according to the previously published calibration procedure [10]. Custom image analysis software was implemented in C++. Mean arterial and venous oxygen saturation values were computed per participant by averaging across the respective vessel segments.

### Reference measurements

For comparison, oxygen saturation was measured using a commercial retinal oximeter (Dynamic Vessel Analyzer/Oximetry Module, Imedos Health GmbH, Jena, Germany). The reference system was equipped with a 3-chip CCD camera, providing separate detection of the color channels and thereby improved spectral separation compared with conventional one-chip Bayer color cameras. Measurements from both systems were compared at the participant level using mean arterial and mean venous saturation values within a circumpapillary annulus with an inner radius of 1 and an outer radius of 1.5 disc diameters. Oxygen saturation was evaluated in all identifiable retinal vessels within this annulus. The quantitative oxygen-saturation calculation was largely automated. Arteries and veins were classified by inspection of the color images, whereas the principal operator-dependent step affecting the quantitative analysis was placement of the analysis annulus centered on the optic disc. Small deviations in annulus placement were expected to have only a minor influence on the overall oxygen-saturation estimate because essentially the same vessel segments would remain included in the analysis. The same vessel segments were analyzed in both imaging modalities, including at least five arteries and five veins per participant and typically seven to nine vessels of each type. No vessel segments were excluded after image acquisition.

### Statistical analysis

Differences between methods were summarized as mean ± standard deviation. Mean absolute differences were calculated as the mean of the absolute individual differences in oxygen saturation between the serial illumination method and the commercial retinal oximeter for each participant. Statistical analyses were performed using IBM SPSS Statistics Version 26.0.0.0. Statistical significance was defined as *p* < 0.05. Linear regression analysis assessed association between methods for arterial and venous oxygen saturation values. Correlation statistics (R, p) and linear regression parameters were calculated. Approximate normality of the regression residuals was assessed using the Shapiro–Wilk test. No significant deviations from normality were detected for either the arterial regression model (W = 0.951, *p* = 0.702) or the venous regression model (W = 0.910, *p* = 0.316). Given the small exploratory sample, the regression analyses were interpreted descriptively. Agreement between methods was assessed using Bland–Altman analysis, including calculation of mean bias and limits of agreement (± 1.96 SD). Potential proportional bias was additionally explored by linear regression of the method difference against the mean of the two measurements.

## Results

All nine participants could be measured using the serial illumination technique.

**Fig. 1:**
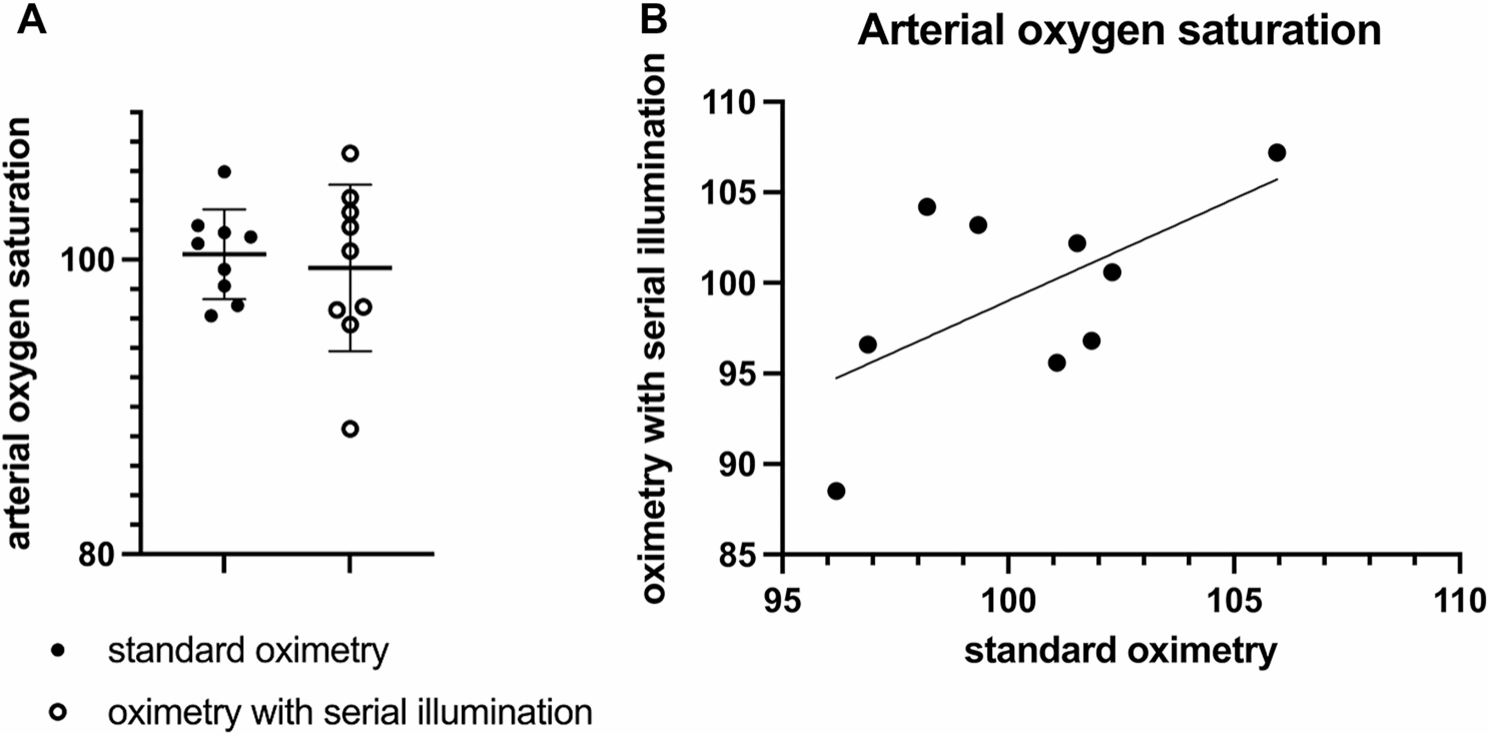
Arterial oxygen saturation measured with standard retinal oximetry and serial illumination retinal oximetry. (**a**) Comparison of mean arterial oxygen saturation values obtained with standard retinal oximetry and serial illumination retinal oximetry. (**b**) Linear regression analysis comparing arterial oxygen saturation values measured with standard retinal oximetry and serial illumination retinal oximetry

Figure 1a shows the comparison of mean arterial oxygen saturation values obtained with standard retinal oximetry and serial illumination retinal oximetry. Mean arterial oxygen saturation was 99.4 ± 5.7% with serial illumination and 100.4 ± 3.0% with the standard oximeter. Arterial oxygen saturation values slightly exceeding 100% are commonly observed in retinal vessel oximetry and reflect calibration characteristics of the optical density ratio rather than physiological oxygen saturation above 100% [5, 10].

Figure 1b shows the corresponding regression analysis for arterial oxygen saturation. Arterial oxygen saturation values showed a borderline non-significant correlation between the two techniques (*R* = 0.606, *p* = 0.084).

**Fig. 2:**
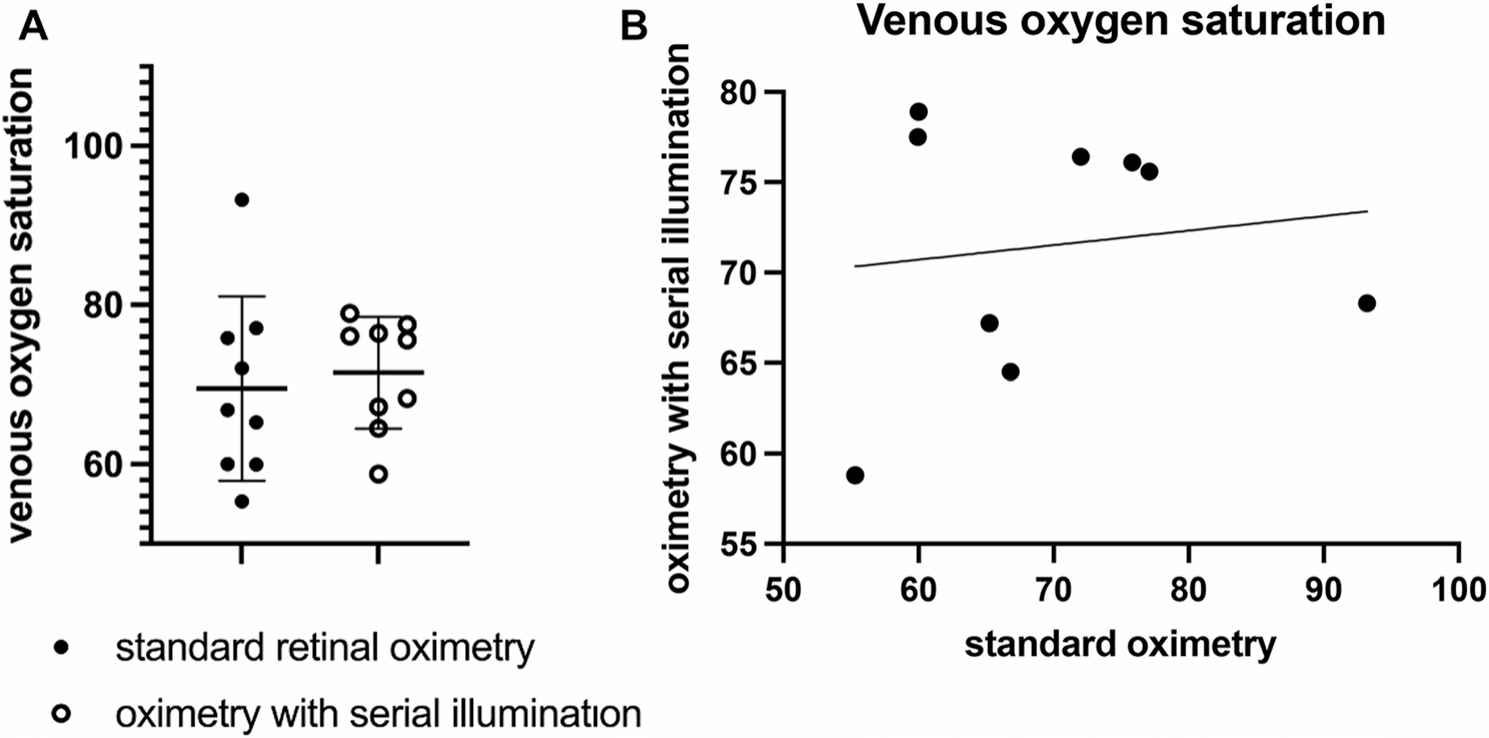
Venous oxygen saturation measured with standard retinal oximetry and serial illumination retinal oximetry. (**a**) Comparison of mean venous oxygen saturation values obtained with standard retinal oximetry and serial illumination retinal oximetry. (**b**) Linear regression analysis comparing venous oxygen saturation values measured with standard retinal oximetry and serial illumination retinal oximetry

Figure 2a shows the comparison of mean venous oxygen saturation values obtained with standard retinal oximetry and serial illumination retinal oximetry. Mean venous oxygen saturation was 71.5 ± 7.0% with serial illumination and 69.5 ± 11.6% with the standard oximeter.

Figure 2b shows the corresponding regression analysis for venous oxygen saturation. No correlation was found between the two techniques.

The mean absolute difference between the two methods was 3.6 ± 2.7% for arteries and 8.4 ± 9.3% for veins.

**Fig. 3:**
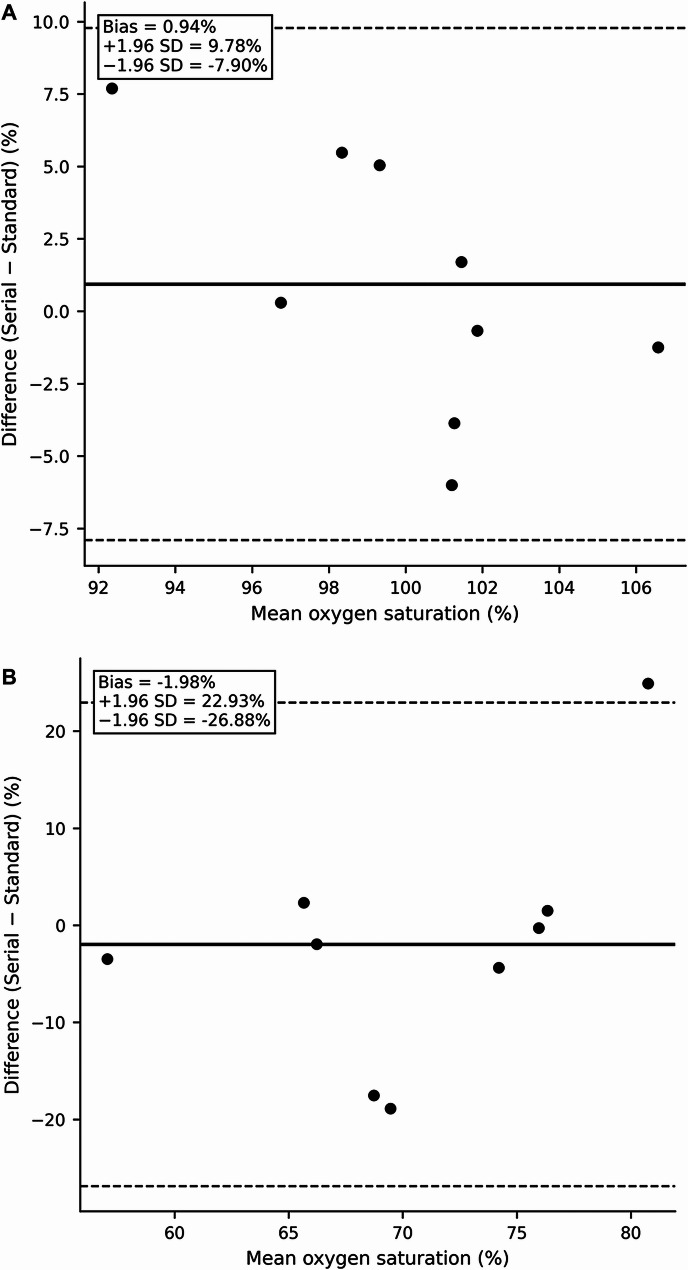
Agreement between standard retinal oximetry and serial illumination retinal oximetry. (**a**) Bland–Altman plot showing agreement for arterial oxygen saturation measurements. (**b**) Bland–Altman plot showing agreement for venous oxygen saturation measurements

Figure 3a shows the Bland–Altman analysis for arterial oxygen saturation. The mean difference (bias) was 0.94%, with limits of agreement ranging from − 7.9% to + 9.8%. This indicates a small average systematic bias, although the limits of agreement demonstrate non-negligible interindividual differences between the methods. Exploratory regression of the arterial method difference against the mean oxygen saturation showed a negative association (*r* = − 0.638), which did not reach statistical significance in the present sample (*p* = 0.065).

Figure 3b shows the Bland–Altman analysis for venous oxygen saturation. The mean bias was − 1.98%, with substantially wider limits of agreement ranging from − 26.9% to + 22.9%, indicating markedly greater variability than observed for arterial measurements.

Overall, arterial measurements demonstrated only a small systematic bias relative to the commercial reference method, whereas venous measurements exhibited substantially greater variability.

## Discussion

This proof-of-concept study demonstrates that serial dual-wavelength illumination can be implemented in a conventional fundus camera and used for retinal vessel oximetry. The central methodological rationale is to avoid detector-level spectral overlap associated with simultaneous dual-wavelength acquisition using one-chip color cameras, which can introduce crosstalk and systematic bias in optical density measurements [8, 10]. By temporally separating illumination wavelengths while maintaining the same optical detection pathway, serial illumination shifts wavelength separation from the detector to the illumination stage, a concept consistent with spectrophotometric foundations of retinal oximetry and sequential spectral imaging approaches in retinal imaging [7, 14].

Previous studies by Kim et al. [15] and Singh et al. [16] demonstrated that retinal oximetry based on nonsimultaneous image acquisition using conventional fundus cameras is feasible. In contrast to these earlier implementations, the present work focuses on a serial dual-wavelength LED-based illumination approach and explicitly compares the resulting oxygen saturation measurements with those obtained from a commercial retinal oximeter. Whereas Kim et al. and Singh et al. primarily emphasized feasibility and repeatability, our study specifically addresses detector-level spectral crosstalk as a motivation for serial illumination and evaluates inter-method agreement against a clinically established commercial system using Bland–Altman analysis.

Arterial oxygen saturation measured with serial illumination (99.4 ± 5.7%) was similar to the commercial oximeter values (100.4 ± 3.0%). The mean absolute difference (3.6 ± 2.7%) is within the magnitude of variability commonly encountered in retinal oximetry when comparing systems or repeating measurements, particularly when algorithms incorporate compensation for vessel diameter and pigmentation [5, 6, 10]. The greater robustness of the arterial measurements observed in the present dataset cannot readily be attributed to higher arterial image contrast. At the oxygen-sensitive wavelength, venous vessels generally exhibit greater contrast than arteries because of the higher absorption of deoxygenated hemoglobin [11]. The greater venous inter-method variability observed here is therefore likely multifactorial.

Bland–Altman analysis provides a more appropriate assessment of agreement than correlation analysis alone [18]. The small arterial bias and moderate limits of agreement observed in this study suggest that serial illumination provides arterial oxygen saturation estimates with only a small systematic bias relative to the reference method. The arterial Bland–Altman plot suggested a negative relationship between measurement difference and mean oxygen saturation. Exploratory analysis confirmed a negative association, although it did not reach statistical significance in the present small sample (*r* = − 0.638, *p* = 0.065). Whether this represents proportional bias therefore remains uncertain and should be evaluated in a larger dataset. The magnitude of variability is comparable to previously reported inter-device differences in retinal oximetry [5, 10]. The repeatability of standard retinal oximetry systems has been reported previously and should be considered when interpreting the observed inter-method variability [5, 10]. The observed inter-method variability should also be considered in relation to the magnitude of oxygen-saturation differences reported under physiological and pathological conditions [4–6].

The observed borderline non-significant correlation should be interpreted cautiously and is plausibly explained by restricted physiological dynamic range in a cohort selected to exclude ocular and systemic vascular disease [5, 6]. In such participants, arterial oxygen saturation tends to cluster near the upper physiological range, limiting between-subject variability. When the dynamic range of the measured values is limited, correlation coefficients may remain modest despite comparable central tendency and dispersion between methods, reflecting the well-recognized limitations of correlation-based method comparison. From a methodological perspective, the more informative point is that the central tendency and dispersion of arterial saturation are comparable between the serial approach and the reference system (Fig. 1a), suggesting that serial illumination can reproduce arterial oxygenation estimates consistent with established dual-wavelength oximetry concepts [7, 8, 10].

In contrast, venous oxygen saturation showed greater dispersion between methods and no correlation. This pattern is consistent with substantial prior experience that venous oximetry is more variable and often less reproducible than arterial oximetry across platforms [5, 6, 10]. Multiple factors can contribute.

One participant showed an unusually high venous oxygen saturation value of 93.2% with the standard oximeter, while the corresponding arterial oxygen saturation was 101.1%. Re-evaluation of the original data confirmed these values and did not identify a transcription or processing error. Because no predefined exclusion criterion for unusually high oximetry values had been specified and no technical error could be identified, the datapoint was retained in the analysis. Given the small sample size, this observation contributes substantially to the observed venous inter-method variability and influences both the regression and Bland–Altman analyses.

First, oxygen-saturation measurements may be sensitive to segmentation and noise-related errors because vessel contrast varies with wavelength and imaging conditions. Optical density estimation depends on accurately defining vessel and adjacent background regions. Errors in background selection, local fundus reflectance heterogeneity, and image noise may therefore influence the calculated optical density ratio [10, 11]. Second, vessel diameter effects and pigmentation confounding are known to influence saturation estimates, and residual unmodeled effects may contribute to inter-method variability [6, 11]. Third, physiological variability may be larger for venous saturation because it reflects oxygen extraction and therefore depends on local metabolic demand, flow, and microvascular regulation, which may fluctuate over short time scales [1–3].

Serial acquisition introduces an additional sensitivity to inter-frame motion. Even after registration, small residual misalignments, especially at vessel edges, can shift pixel sampling between vessel lumen and background and thereby alter optical density ratios. However, the extent to which residual registration errors contributed specifically to the greater venous variability observed in the present dataset cannot be determined. Importantly, this does not indicate that serial illumination is unsuitable. Rather, it identifies the technical aspects that must be optimized (acquisition timing, registration quality, segmentation robustness) to improve venous stability, consistent with known bottlenecks in retinal oximetry method development [5, 6, 10]. In the present dataset, image registration was successful in all cases, and no clinically relevant motion blur was observed that impaired vessel segmentation or oxygen saturation analysis, despite the 250 ms exposure time per wavelength. Image quality was considered sufficient for vessel segmentation and oxygen saturation analysis in all included cases.

Most methodological refinement in retinal oximetry has historically focused on algorithmic compensation—vessel diameter correction, pigmentation normalization, calibration procedures, and segmentation automation [5, 11]. Serial illumination complements these approaches by addressing spectral purity at acquisition rather than in post-processing, directly avoiding detector channel overlap effects that cannot be fully eliminated by software corrections. This is particularly relevant when adapting oximetry to different cameras/sensors or when aiming to use monochrome sensors with higher sensitivity and potentially improved noise characteristics, a direction that has been discussed in broader multispectral/hyperspectral retinal imaging research [14].

Dedicated retinal imaging systems based on monochrome cameras and narrow band-pass filters represent an established solution for achieving high spectral separation while avoiding detector channel overlap [12, 13]. Such systems, however, require dedicated imaging hardware and optical filter arrangements. In contrast, the present approach is not intended to replace monochrome imaging systems but to provide a technically simple alternative that can be integrated into conventional color fundus cameras. By shifting wavelength separation from the detector to the illumination stage, serial illumination mitigates detector-level spectral crosstalk without requiring major modifications of the imaging sensor.

However, serial illumination also imposes engineering requirements: good compensation of chromatic aberrations in the optics, faster effective acquisition (or better motion management), and robust registration. The relatively narrow spectral bandwidth (2.5 nm) was chosen to ensure high spectral specificity and minimize overlap between the absorption characteristics of oxy- and deoxyhemoglobin. While broader bandwidths could increase photon flux and thereby reduce acquisition time, they would also increase spectral overlap between oxy- and deoxyhemoglobin absorption characteristics, thereby reducing sensitivity of the optical density ratio. Future iterations may therefore investigate the trade-off between spectral bandwidth, signal intensity, acquisition speed, and motion sensitivity, particularly in relation to pupil reflex dynamics and inter-frame eye movement. Likewise, further automation of operator-dependent analysis steps may reduce analysis-related variability and improve inter-study comparability. These steps are typical in the maturation pathway of imaging-based biomarkers and are consistent with prior development trajectories in retinal oximetry [5, 10].

The exclusion criteria (retinal disease, glaucoma, diabetes, cardiovascular disease) were chosen to minimize confounding and to test the acquisition/analysis pipeline under relatively homogeneous physiological conditions. This is appropriate for a feasibility and technical comparability study. Nevertheless, it limits dynamic range and therefore limits the strength of correlation-based method comparisons. A logical next step is validation in cohorts with broader oxygenation variability (e.g., controlled hypoxia paradigms or disease cohorts), where method association can be assessed across a wider range and where clinical questions become relevant. Such studies should also incorporate repeatability assessments (test–retest) because reproducibility is central for translation of oximetry into clinical research endpoints.

## Limitations

This study has several limitations. The sample size (*n* = 9) is small, and the design is exploratory, limiting inferential conclusions. The limited sample size also reduced statistical power for correlation analyses. Consequently, the Bland–Altman bias and limits of agreement should be interpreted cautiously, as confidence intervals around these estimates are expected to be relatively wide in such a small exploratory dataset. Larger validation studies will be required to establish the precision and generalizability of the observed agreement.

No dedicated test–retest reproducibility assessment was performed. Although no formal inter-observer reproducibility analysis was conducted, the quantitative oxygen-saturation calculation was largely automated. Operator-dependent steps included classification of arteries and veins by inspection of the color images and placement of the analysis annulus around the optic disc, with annulus placement representing the principal operator-dependent step affecting the quantitative analysis. Future validation studies should nevertheless include dedicated reproducibility analyses.

A further limitation of sequential acquisition is the potential influence of pulsatile changes in vessel diameter between the two wavelength recordings. Assuming a diameter variation of approximately 6%, a vessel with a diameter of 150 μm would change by approximately 9 μm. Based on the previously reported dependence of calculated oxygen saturation on vessel diameter and the corresponding compensation procedure [10], this would correspond to an estimated oxygen-saturation difference of approximately 2.34%. This is of the same order of magnitude as the reported reproducibility of 2.52% for arterial and 3.25% for venous oxygen-saturation measurements using this approach [10]. Nevertheless, cardiac-cycle-related diameter changes represent a potential source of variability specific to serial acquisition.

Prior studies on nonsimultaneous retinal oximetry have reported repeatability in the range of approximately 2–5% for arterial and venous measurements [15, 16]. The relative contributions of inter-method differences, sequential acquisition, and intrinsic repeatability cannot be determined from the present dataset.

A further limitation is the absence of an independent in vivo gold standard for retinal vessel oxygen saturation. Systemic arterial and venous blood gas measurements are invasive and do not directly represent oxygen saturation within individual retinal vessels. The present study therefore evaluated agreement with an established commercial retinal oximeter rather than absolute accuracy. Future validation may require complementary physiological calibration approaches or controlled oxygenation protocols.

## Conclusions

Serial dual-wavelength illumination is feasible for retinal vessel oximetry in a conventional fundus camera and addresses detector-level spectral overlap inherent to simultaneous dual-wavelength acquisition with one-chip color cameras. In this proof-of-concept cohort, arterial oxygen saturation measurements demonstrated only a small systematic bias relative to the commercial reference system, whereas venous measurements showed greater variability. The approach provides a flexible technical platform for further optimization and for future validation studies emphasizing repeatability, motion sensitivity, and performance in cohorts with broader hemoglobin oxygenation variability. The approach may facilitate broader clinical accessibility of retinal oximetry by reducing hardware complexity while directly addressing detector-level spectral limitations.

## Data Availability

The datasets generated and/or analyzed during the current study are available from the corresponding author on reasonable request.

## References

[1] Yu DY, Cringle SJ. Oxygen distribution and consumption within the retina in vascularised and avascular retinas and in animal models of retinal disease. Prog Retin Eye Res. 2001;20(2):175–208. 11173251 10.1016/s1350-9462(00)00027-6

[2] Stefansson E. Oxygen and diabetic eye disease. Graefes Arch Clin Exp Ophthalmol. 1990;228(2):120–3. 2186971 10.1007/BF00935719

[3] Pournaras CJ, Rungger-Brändle E, Riva CE, Hardarson SH, Stefansson E. Regulation of retinal blood flow in health and disease. Prog Retin Eye Res. 2008;27(3):284–330. 18448380 10.1016/j.preteyeres.2008.02.002

[4] Hardarson SH, Stefánsson E. Retinal oxygen saturation is altered in diabetic retinopathy. Br J Ophthalmol. 2012;96(4):560–3. 22080478 10.1136/bjophthalmol-2011-300640

[5] Hardarson SH, Harris A, Karlsson RA, Halldorsson GH, Kagemann L, Rechtman E, Zoega GMr, Eysteinsson T, Benediktsson JA, Thorsteinsson A, et al. Automatic Retinal Oximetry. Investig Ophthalmol Vis Sci. 2006;47(11):5011–6. 17065521 10.1167/iovs.06-0039

[6] Geirsdottir A, Hardarson SH, Olafsdottir OB, Stefánsson E. Retinal oxygen metabolism in exudative age-related macular degeneration. Acta Ophthalmol. 2014;92(1):27–33. 24447786 10.1111/aos.12294

[7] Delori FC. Noninvasive technique for oximetry of blood in retinal vessels. Appl Opt. 1988;27(6):1113–25. 20531526 10.1364/AO.27.001113

[8] Smith MH, Denninghoff KR, Lompado A, Hillman LW. Effect of multiple light paths on retinal vessel oximetry. Appl Opt. 2000;39(7):1183–93. 18338002 10.1364/ao.39.001183

[9] Schweitzer D, Hammer M, Kraft J, Thamm E, Königsdörffer E, Strobel J. In vivo measurement of the oxygen saturation of retinal vessels in healthy volunteers. IEEE Trans Biomed Eng. 1999;46(12):1454–65. 10612903 10.1109/10.804573

[10] Hammer M, Vilser W, Riemer T, Schweitzer D. Retinal vessel oximetry-calibration, compensation for vessel diameter and fundus pigmentation, and reproducibility. J Biomed Opt. 2008;13(5):054015. 19021395 10.1117/1.2976032

[11] Beach JM, Schwenzer KJ, Srinivas S, Kim D, Tiedeman JS. Oximetry of retinal vessels by dual-wavelength imaging: calibration and influence of pigmentation. J Appl Physiol (1985). 1999;86(2):748–58. 9931217 10.1152/jappl.1999.86.2.748

[12] Johnson WR, Wilson DW, Fink W, Humayun M, Bearman G. Snapshot hyperspectral imaging in ophthalmology. J Biomed Opt. 2007;12(1):014036. 17343511 10.1117/1.2434950

[13] Felder AE, Wanek J, Blair NP, Shahidi M. Inner Retinal Oxygen Extraction Fraction in Response to Light Flicker Stimulation in Humans. Invest Ophthalmol Vis Sci. 2015;56(11):6633–7. 26469748 10.1167/iovs.15-17321 PMC4611954

[14] Kagemann L, Wollstein G, Wojtkowski M, Ishikawa H, Townsend KA, Gabriele ML, Srinivasan VJ, Fujimoto JG, Schuman JS. Spectral oximetry assessed with high-speed ultra-high-resolution optical coherence tomography. J Biomed Opt. 2007;12(4):041212. 17867801 10.1117/1.2772655 PMC2916162

[15] Kim S, Kim D, Suh M, Kim M, Kim HC. Retinal Oximetry Based on Nonsimultaneous Image Acquisition Using a Conventional Fundus Camera. IEEE Trans Med Imaging. 2011;30:1577–80. 21478073 10.1109/TMI.2011.2140329

[16] Singh S, Velu G, Raman R. Development and Validation of Non simultaneous Retinal Image Acquisition–Based Retinal Oximeter. Sci Rep. 2017;7(1):4270. 28655932 10.1038/s41598-017-04085-x PMC5487317

[17] Garhofer G, Bek T, Boehm AG, Gherghel D, Grunwald J, Jeppesen P, Kergoat H, Kotliar K, Lanzl I, Lovasik JV, et al. Use of the retinal vessel analyzer in ocular blood flow research. Acta Ophthalmol. 2010;88(7):717–22. 19681764 10.1111/j.1755-3768.2009.01587.x

[18] Bland JM, Altman DG. Statistical methods for assessing agreement between two methods of clinical measurement. Lancet (London England). 1986;1(8476):307–10. 2868172

